# Ratiometric and discriminative visualization of autophagic processes with a novel dual-responded lysosome-specific fluorescent probe

**DOI:** 10.1186/s40824-023-00409-3

**Published:** 2023-07-06

**Authors:** Fan Zheng, Yeshuo Ma, Jipeng Ding, Shuai Huang, Shengwang Zhang, Xueyan Huang, Bin Feng, Hongliang Zeng, Fei Chen, Wenbin Zeng

**Affiliations:** 1 Xiangya School of Pharmaceutical Sciences, Central South University, Changsha, 410013 People’s Republic of China; 2 Hunan Key Laboratory of Diagnostic and Therapeutic Drug Research for Chronic Diseases, Central South University, Changsha, 410013 People’s Republic of China; 3 The Third Xiangya Hospital, Central South University, Changsha, People’s Republic of China; 4 Research Institute of Chinese Medicine, Hunan Academy of Chinese Medicine, Changsha, 410013 People’s Republic of China

**Keywords:** Autophagy visualization, Fluorescent probe, Lysosome-specific, Ratiometric imaging, Dual-responsive, pH, Viscosity, Acetaminophen-induced liver injury

## Abstract

**Background:**

Autophagy is a critical self-eating pathway involved in numerous physiological and pathological processes. Lysosomal degradation of dysfunctional organelles and invading microorganisms is central to the autophagy mechanism and essential for combating disease-related conditions. Therefore, monitoring fluctuations in the lysosomal microenvironment is vital for tracking the dynamic process of autophagy. Although much effort has been put into designing probes for measuring lysosomal viscosity or pH separately, there is a need to validate the concurrent imaging of the two elements to enhance the understanding of the dynamic progression of autophagy.

**Methods:**

Probe **HFI** was synthesized in three steps and was developed to visualize changes in viscosity and pH within lysosomes for real-time autophagy tracking. Then, the spectrometric determination was carried out. Next, the probe was applied to image autophagy in cells under nutrient-deprivation or external stress. Additionally, the performance of **HFI** to monitor autophagy was employed to evaluate acetaminophen-induced liver injury.

**Results:**

We constructed a ratiometric dual-responsive probe, **HFI**, with a large Stokes shift over 200 nm, dual-wavelength emission, and small background interference. The ratiometric fluorescent signal (R = *I*_610_/*I*_460_) of **HFI** had an excellent correlation with both viscosity and pH. More importantly, high viscosity and low pH had a synergistic promotion effect on the emission intensity of **HFI**, which enabled it to specially lit lysosomes without disturbing the inherent microenvironment. We then successfully used **HFI** to monitor intracellular autophagy induced by starvation or drugs in real-time. Interestingly, **HFI** also enabled us to visualize the occurrence of autophagy in the liver tissue of a DILI model, as well as the reversible effect of hepatoprotective drugs on this event.

**Conclusions:**

In this study, we developed the first ratiometric dual-responsive fluorescent probe, **HFI**, for real-time revealing autophagic details. It could image lysosomes with minimal perturbation to their inherent pH, allowing us to track changes in lysosomal viscosity and pH in living cells. Ultimately, **HFI** has great potential to serve as a useful indicator for autophagic changes in viscosity and pH in complex biological samples and can also be used to assess drug safety.

**Supplementary Information:**

The online version contains supplementary material available at 10.1186/s40824-023-00409-3.

## Introduction

Autophagy is a highly conserved cellular process that involves the degradation and recycling of various cellular components to provide nutrients for cells [1–3]. It plays a critical role in maintaining cellular homeostasis, metabolism, and energy renewal, as well as in processes such as resistance to starvation, elimination of invading microorganisms, and regulation of immunity [4–7]. Dysregulation of autophagy has been linked to a variety of diseases, including cancers, liver injury, diabetes, neurodegenerative disorders, and infectious diseases [8–11]. However, the precise changes in autophagy in various diseases have not been fully elucidated [12]. Therefore, there is a growing need to monitor the dynamic process of autophagy in detail for a better understanding of the underlying pathological mechanisms. So far, transmission electron microscopy, western blot (WB) (Atg8/LC3), and GFP-Atg8/LC3 fluorescence microscopy are typically involved in the detection of autophagy [13, 14]. Nevertheless, these methods are not only time-consuming but also lack single-cell resolution. Under this circumstance, the development of efficient and precise approaches to monitor the dynamic process of autophagy in real-time is still highly required. In this predicament, fluorescent probes have provided a simple, noninvasive, and sensitive platform for imaging in living biological samples [15–20].

The general mechanism of autophagy involves the delivering of dysfunctional organelles or unnecessary cellular components to autophagic vesicles, called autophagosomes, followed by the fusion of autophagosomes with lysosomes to form autolysosomes to retrieve the recyclable contents [21, 22]. Although there are three distinct forms of autophagy based on different cargo delivery intermediaries, the damaged cytoplasmic cargoes are all delivered into lysosomes eventually for degradation [23]. On account of the essential role of those lysosomal degrading enzymes in autophagy, creating fluorescent probes to monitor changes in the lysosomal microenvironment is critical for understanding the autophagy mechanism and advancing research on related diseases [24–26]. Specifically, visualizing the changes in viscosity and pH in lysosomes is significant, as the function of lysosomes can be reflected by viscosity, while the activity of lysosomal hydrolases is closely related to pH [23, 27–29]. Although much effort has been put into designing probes for measuring lysosomal either viscosity or pH separately, there is still a significant need to validate the concurrent imaging of the two elements to enhance the understanding of the dynamic progression of autophagy (Table S1).

**Scheme. 1:**
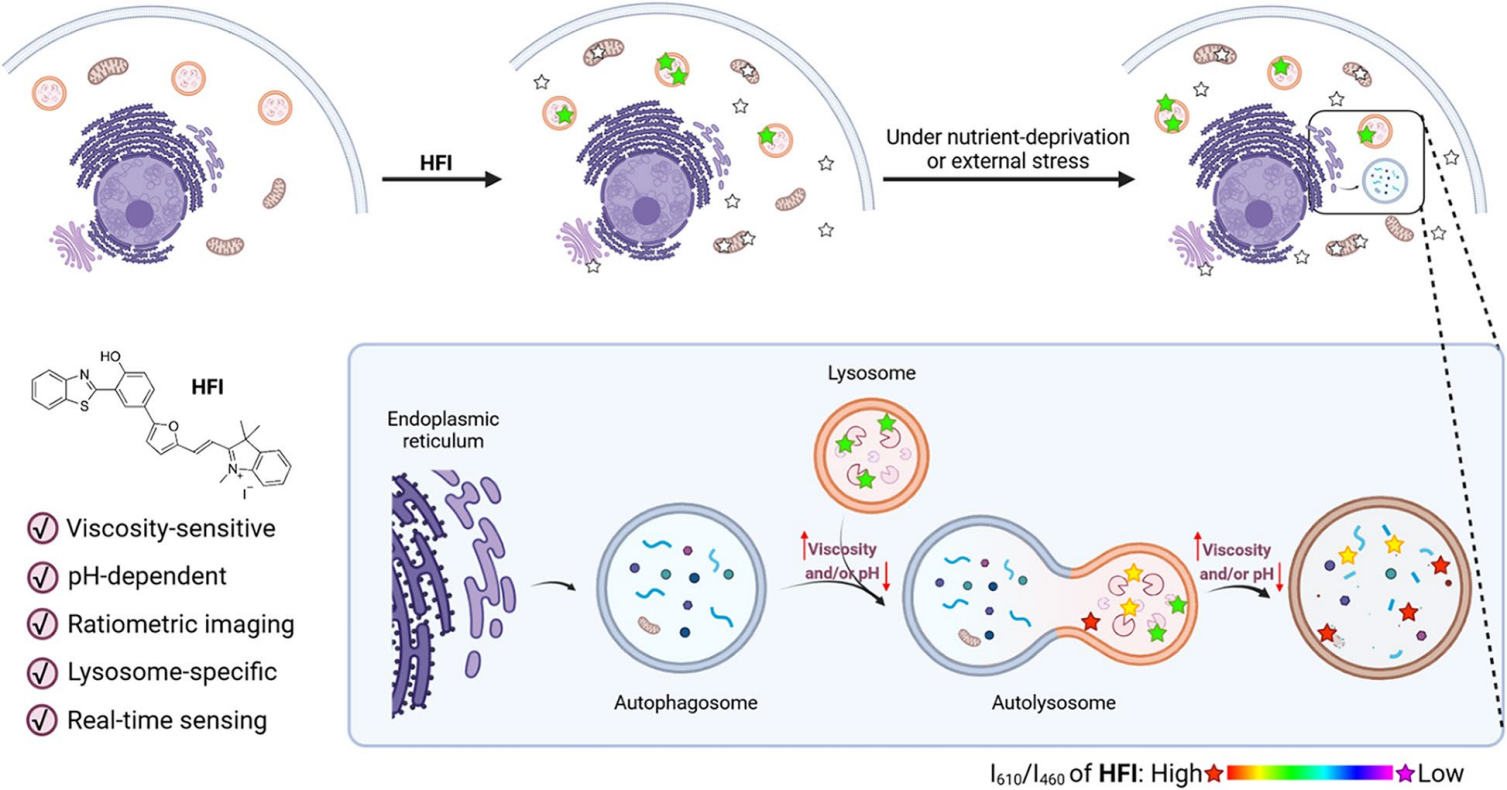
Schematic illustration of **HFI** for real-time monitoring viscosity and pH fluctuations in lysosomes during autophagy. Compared with other organelles, only lysosomes with highly viscous and acidic microenvironments could be specifically lit by probe **HFI**. Once facing nutrient-deprivation or external stress, the ratiometric fluorescent signal (R = *I*_610_/*I*_460_) of **HFI** would raise with time to reveal the autophagic details

Herein, we developed the first ratiometric probe, 2-(2-(5-(3-(benzo[d]thiazol-2-yl)-4-hydroxyphenyl)furan-2-yl)vinyl)-1,3,3-trimethyl-3H-indol-1-ium iodide (**HFI**), with the merits of large Stokes shift, dual-wavelength emission, and small background interference, for simultaneously tracking the changes of both viscosity and pH in lysosomes during autophagy. The hydroxyl group on **HFI** could serve as a pH-dependent group, while the free intramolecular rotation of the probe could be limited to sense viscosity. As illustrated in Scheme 1, it was interesting to find that **HFI** could image lysosomes without involving weakly basic nitrogen-containing side chains (i.e., morpholine or N, N-dimethylethylenediamine) in the structure [30]. In this perspective, the inherent lysosomal pH would hardly be perturbed by **HFI** as it was often increased by many organic amines-based pH probes (an alkalinizing effect) [30, 31]. Then, **HFI** was applied to monitor autophagy under nutrient-deprivation or external stresses by concurrently imaging the variations of viscosity and pH. More importantly, **HFI** has been employed to reveal the details of autophagy in acetaminophen (APAP)-induced liver injury, representing the potential of this probe in auxiliary assessing drug safety.

## Results

### Design and synthesis of HFI

**HFI** was successfully synthesized in three steps reaction through the conjugated connection of an excited-state intramolecular proton transfer (ESIPT)-fluorophore 2-(2’-hydroxy-phenyl)benzothiazole (HBT) as an electron donor with an indolium iodide as electron acceptor. Meanwhile, the hydroxyl group of the HBT skeleton could serve as a pH-dependent group by influencing tautomerization upon excitation (Scheme S1). Based on the twisted intramolecular charge transfer (TICT) effect, the free intramolecular rotation of **HFI **could be limited under high viscosity to lead to a significant fluorescence enhancement. The chemical structure of **HFI** was characterized by NMR and HRMS analysis, as shown in the Supporting Information (Fig. S1-S3).

### Spectroscopic properties of HFI

**Fig. 1:**
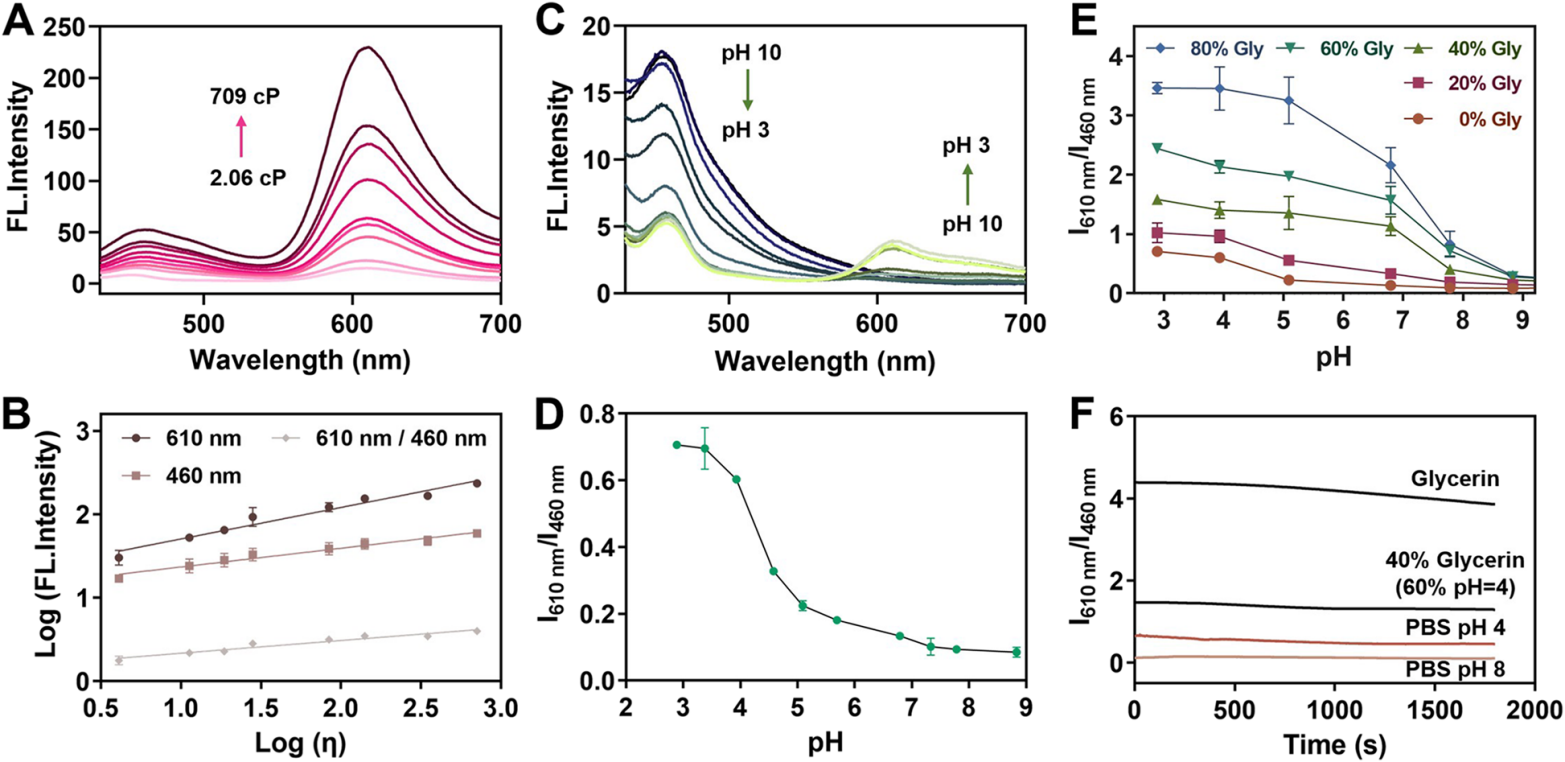
Spectroscopic properties of **HFI**. **A** Fluorescence spectra of **HFI** (10 μM) in methanol containing increasing concentrations of glycerol. **B** Dependence of Log(I) on Log(η). Log(*I*_610_) = 0.3777*Log(η) + 1.328 (R^2^ = 0.9609), Log(*I*_460_) = 0.2250*Log(η) + 1.144 (R^2^ = 0.9669), Log(*I*_610_/*I*_460_) = 0.1527*Log(η) + 0.1834 (R^2^ = 0.9400). **C** Emission spectra of the probe in PBS buffer with different pH values (3–10). **D** Plots of the *I*_610_/*I*_460_ versus pH. **E** Plots of *I*_610_/*I*_460_ versus pH in various proportions of glycerin and different pH PBS buffer. **F** Photostability experiments of **HFI** (10 μM) in PBS buffer (pH 4 or pH 8), PBS buffer (pH 4) / glycerin (2/5, v/v), or glycerin under continuous irradiation by laser light. *λ*_ex_ = 400 nm

First, the photophysical properties of **HFI** were investigated in different solvents. As depicted in Fig. S4A-B, few changes were observed in the absorption of **HFI**, while the fluorescence intensity in glycerin was several times stronger than that in the other solvents. Under this circumstance, we further provided the quantitative evaluation of **HFI** against viscosity in different proportions of methanol and glycerol from 2.06 cP (100% methanol) to 709 cP (100% glycerol). As shown in Fig. 1A, the fluorescence intensity of the probe at 610 nm remarkably raised about 15-fold with the fraction of glycerol from 0 to 100%. Importantly, the fluorescence intensity at 610 nm, 460 nm, and the ratio between them (*I*_610_/*I*_460_) all showed good linear relationships with viscosity (Fig. 1B). These results indicated the capability of **HFI** for quantitatively monitoring viscosity.

Next, we investigated the response of **HFI** to pH in phosphate buffer solution (PBS) with different pH values. With pH decreasing from 10 to 3, the fluorescence intensity at 460 nm reduced gradually while the emission at 610 nm increased (Fig. 1C). As illustrated in Fig. 1D, the pH value could easily be determined based on the ratiometric fluorescence *I*_610_/*I*_460_, revealing the potential use of **HFI** for the bioimaging of pH fluctuation. Subsequently, the concurrent imaging potential towards viscosity and pH was studied (Fig. 1E). As the viscosity was kept constant, *I*_610_/*I*_460_ showed a negative correlation with the pH values. While the pH remained unchanged, it was shown that with the increase of viscosity, *I*_610_/*I*_460_ intensively increased only under acidic conditions. These results suggested that high viscosity and low pH constituted a synergistic promotion effect on the emission intensity of **HFI**. Moreover, the superior photostability of **HFI** was observed in acidic and basic media, as well as in high-viscosity solutions, which was advantageous for long-term monitoring of the variations of pH and viscosity (Fig. 1F).

### Monitoring the variations of lysosomal viscosity and pH in living cells

Since the cellular microenvironment is complex, interference from other biological molecules should be taken into account before cell imaging. As shown in Fig. S5, no obvious changes in *I*_610_/*I*_460_ were recorded after the addition of the relevant species.

**Fig. 2:**
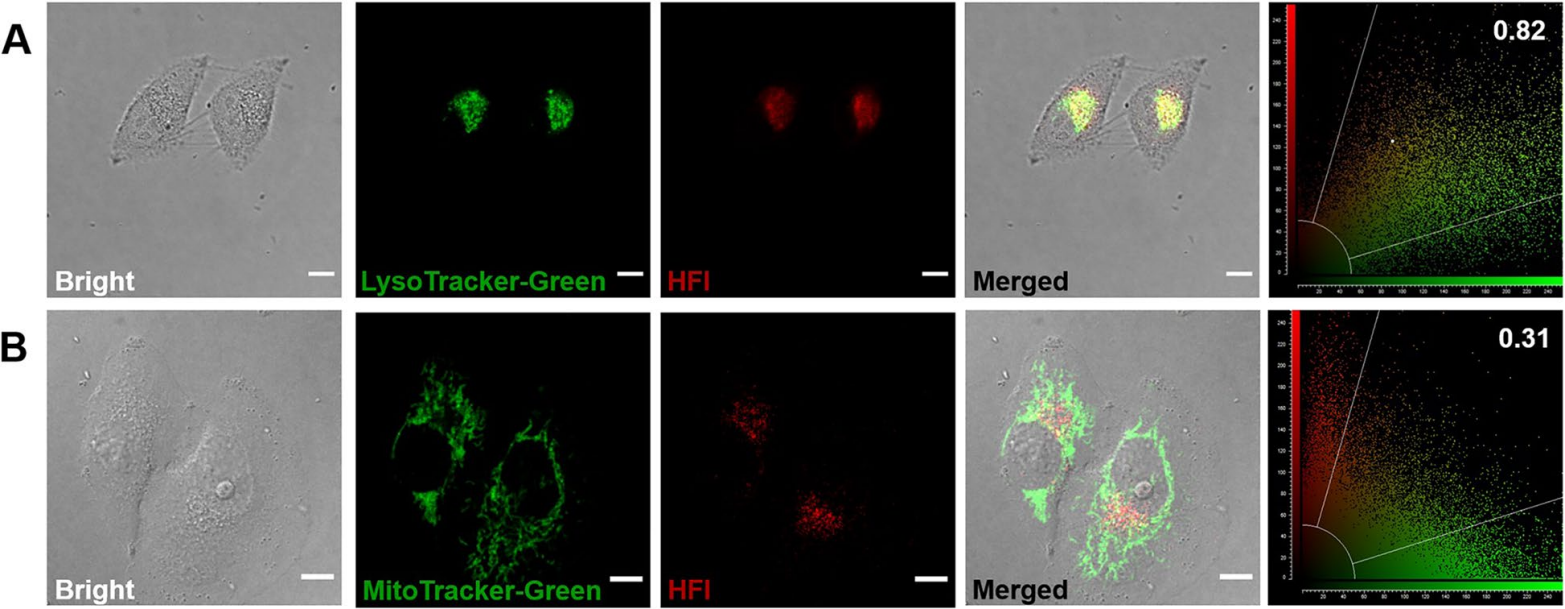
Subcellular co-localization images of **HFI** with LysoTracker-Green (**A**) and MitoTracker-Green (**B**) in HeLa cells. Green channel for LysoTracker-Green and MitoTracker-Green: *λ*_em_ = 525 ± 20 nm, *λ*_ex_ = 488 nm. Red channel for **HFI**: *λ*_em_ = 610 ± 20 nm, *λ*_ex_ = 405 nm. Scale bar, 10 μm

Based on the favorable properties of **HFI** in vitro, its intracellular imaging capacity was further evaluated. First, the biocompatibility of **HFI** was investigated. A standard MTT assay was employed for assessing the cytotoxicity of **HFI** (Fig. S6). Apparently, the survival of HeLa cells still exceeded 80% at concentrations up to 30 μM. The results demonstrated that the probe was negligible toxic to live cells. A co-localization imaging experiment was then carried out to examine the subcellular distribution of **HFI**. Following pretreatment with the probe, HeLa cells were incubated with commercial co-localization dyes, LysoTracker-Green and MitoTracker-Green, respectively. As can be seen from Fig. 2, the red fluorescence of **HFI** was observed a distinct overlap with the green fluorescence of LysoTracker-Green, with a Pearson’s coefficient of 0.82. Conversely, the Pearson’s correlation coefficient in mitochondria was 0.31. These results manifested the predominant lysosomal-imaging capability of **HFI**. It could be explained based on the response characteristic of the probe. It was illustrated that the fluorescence of **HFI** at 610 nm could be intensively activated by the combined effect of high viscosity and low pH (Fig. S7A). Hence, only lysosomes could be specially lit by the probe due to their highly viscous and acidic microenvironment, which provided a basis for **HFI** to track autophagy in lysosomes [32, 33].

**Fig. 3:**
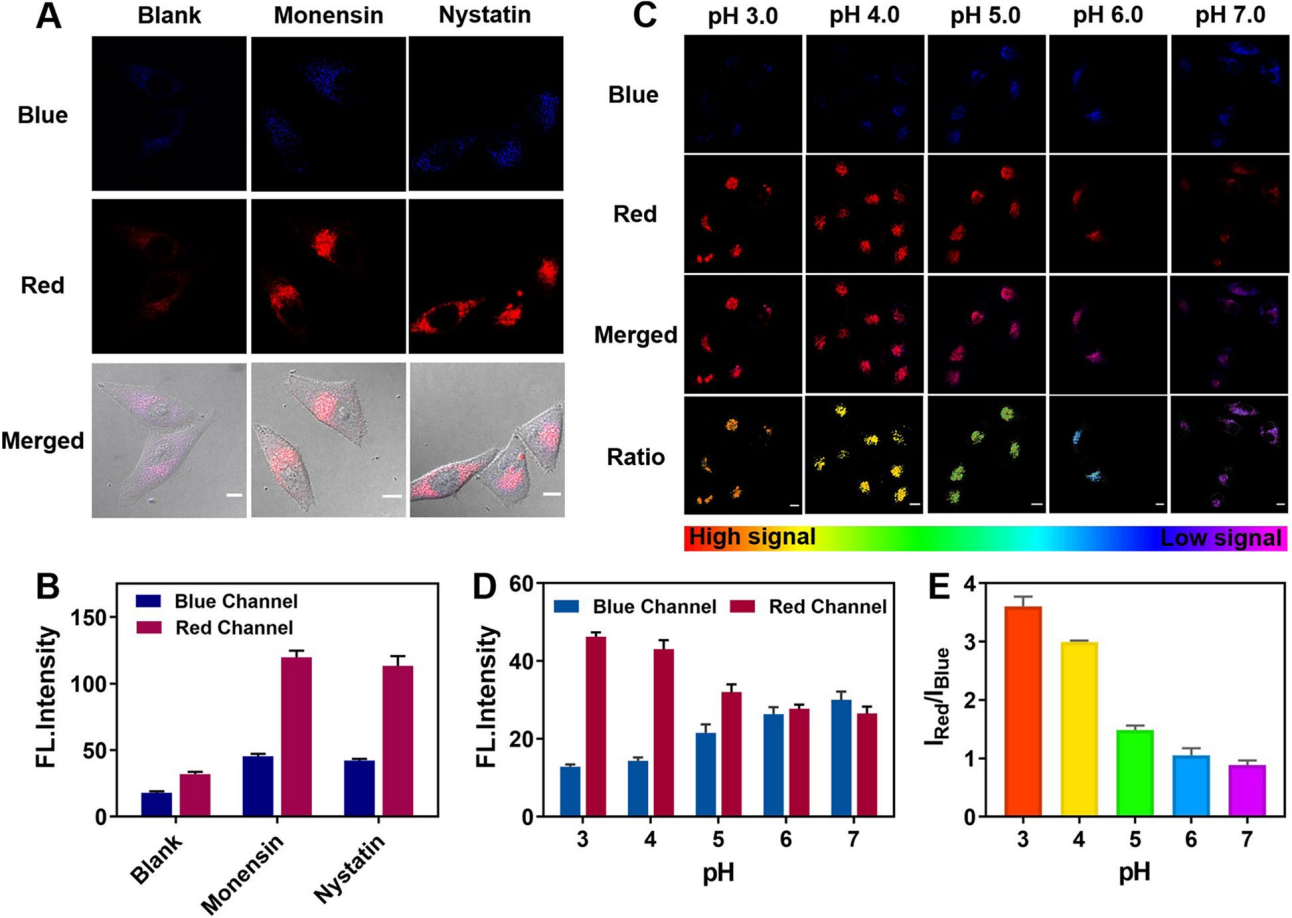
Fluorescence imaging of intracellular viscosity or pH changes by **HFI**. **A** Fluorescence images of HeLa cells pretreated with **HFI** (10 μM) for 30 min and then incubated with monensin (10 μM) or nystatin (10 μM) for another 30 min. **B** Relative fluorescence intensity for **A**. **C** Fluorescence images of **HFI** (10 μM) stained HeLa cells in pH 3.0, 4.0, 5.0, 6.0, and 7.0, respectively. **D** Relative fluorescence intensity for **C**. **E** The intensity ratio of the red channel to the blue channel at different pH values. Blue channel: *λ*_em_ = 460 ± 20 nm, *λ*_ex_ = 405 nm. Red channel: *λ*_em_ = 610 ± 20 nm, *λ*_ex_ = 405 nm. Scale bar, 10 μm

Inspired by the remarkable visualizing potential of **HFI** towards lysosomes, the feasibility of the probe in monitoring the intracellular fluctuations of viscosity and pH was separately evaluated. We initially investigated the function of **HFI** for imaging the viscosity changes in living cells. It is well known that two commonly used antifungal drugs, monensin and nystatin, can selectively transport Na^+^ across the membrane, resulting in cell dehydration and viscosity increasement [34, 35]. Prior to bioimaging, we confirmed that the fluorescence of **HFI** was significantly stable coexisting with monensin or nystatin in vitro (Fig. S7B). As depicted in Fig. 3A-B, HeLa cells treated with monensin or nystatin showed an obviously enhanced fluorescence compared with the control group. This suggested that **HFI** could be employed for the detection of endogenously induced viscosity variations. Then, the pH-dependent performance of **HFI** was determined in HeLa cells as well. The intracellular pH calibration was conducted with high K^+^ buffer solutions to homogenize pH to specific values [36]. As displayed in Fig. 3C-E, the fluorescence intensity in the red channel gradually raised as pH declined from 7.0 to 3.0, whereas that in the blue channel diminished in correspondence, allowing monitoring pH in a ratiometric mode by **HFI**. Overall, the high potential of **HFI** has been validated in tracking the dynamic process of viscosity and pH when lysosomes undergo physiological abnormalities.

### Imaging of lysosomal viscosity and pH changes in starvation and drug-induced autophagy

**Fig. 4:**
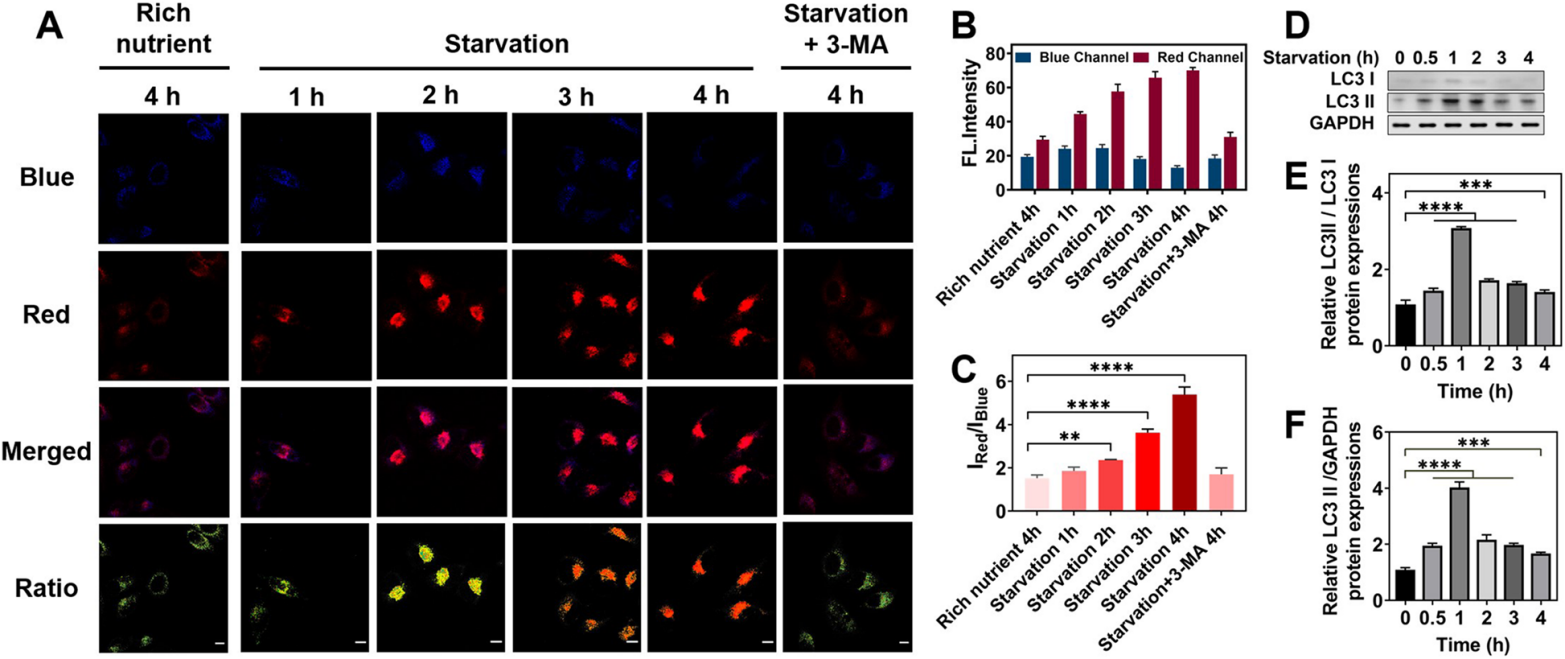
Monitoring the autophagy process of HeLa cells treated with **HFI** under starvation conditions. **A** Confocal fluorescence images of **HFI** (10 μM) stained HeLa cells in 10% FBS-contained DMEM for 4 h, in HBSS for 0–4 h, and in HBSS and 3-MA (300 μM) for 4 h. **B** Relative fluorescence intensity for **A**. **C** The intensity ratio of the red channel to the blue channel at different starvation times. **D** Immunoblotting for LC3 I and LC3 II in HeLa cells incubated in HBSS for 0–4 h. **E** Relative LC3 II / LC3 I protein expressions. **F** Relative LC3 II / GAPDH protein expressions. Blue channel: *λ*_em_ = 460 ± 20 nm, *λ*_ex_ = 405 nm. Red channel: *λ*_em_ = 610 ± 20 nm, *λ*_ex_ = 405 nm. Data are presented as mean ± s.d. (*n* = 3). ** *p* < 0.01, *** *p* < 0.001, and **** *p* < 0.0001. Scale bar, 10 μm

As reported, in the process of autophagy, autophagosomes will finally fuse with lysosomes to form autolysosomes for material reutilization [25]. Considering the distinct internal microenvironment between autophagosomes and lysosomes, we anticipated that both viscosity and pH in lysosomes would be changed during autophagy. To verify this hypothesis, HeLa cells were treated with **HFI** in Hank’s Balanced Salt Solution (HBSS) to build an autophagic model under starvation conditions. The model was successfully established proved by the WB results about the expression of LC3 I and LC3 II (Fig. 4D-F) [37]. As illustrated in Fig. 4A-B, the red fluorescence intensity enhanced sharply till 3 h, and then slowly increased. Meanwhile, the fluorescence in the blue channel increased gently at the first hour in HBSS and remained stable during the next hour, followed by a decrement at 2–4 h. These results revealed a dramatic increase of viscosity at the initial three hours and a significant reduction of pH in the last three hours during this nutrient-deprivation process. It was reflected more distinctly based on the ratio images. The increasing rate of *I*_Red_/*I*_Blue_ tended to raise with the starvation time (Fig. 4C). For comparison, 3-methyladenine (3-MA), an autophagy inhibitor, was performed with the cells incubated in HBSS. As shown in Fig. S8 and Fig. 4A-C, no evident fluorescence fluctuations were observed due to the efficient suppression of autophagy. Consequently, the lysosomal trails of viscosity and pH did change during autophagy which could be real-time visualized by **HFI**.

**Fig. 5:**
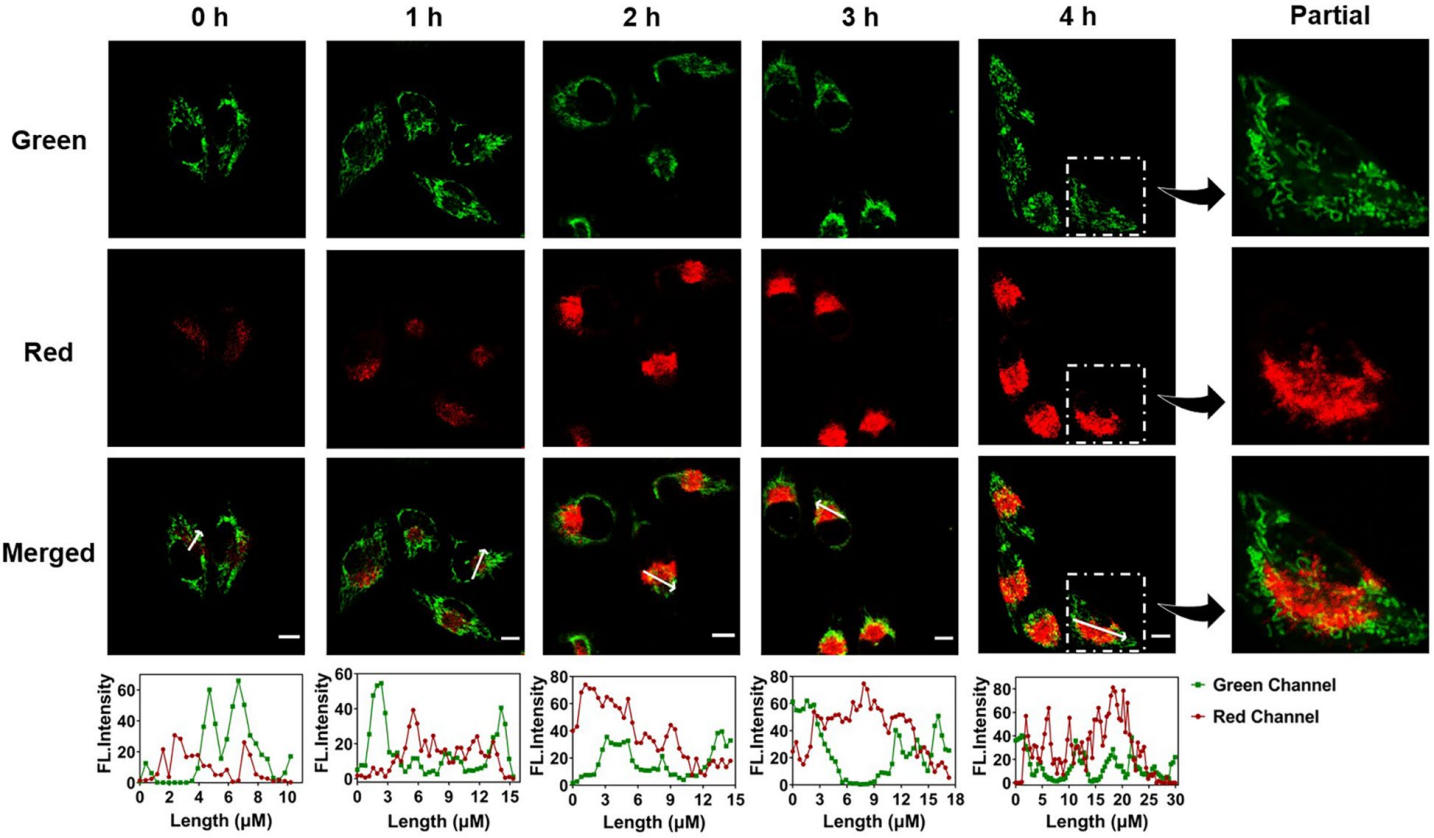
Confocal images of HeLa cells incubated with **HFI** and MitoTracker-Green in HBSS for 0–4 h. Green channel for MitoTracker-Green: *λ*_em_ = 525 ± 20 nm, *λ*_ex_ = 488 nm. Red channel for **HFI**: *λ*_em_ = 610 ± 20 nm, *λ*_ex_ = 405 nm. Scale bar, 10 μm

Moreover, it had been proved that dysfunctional mitochondria would cause autophagy to control mitochondrial quality and quantity [38]. That is to say, an abnormal level of damaged mitochondria might lead to various pathological issues. Thereby, it was of great significance to reveal the autophagic details during this process for a better understanding of the mechanism of the related diseases. Since the ability of **HFI** to real-time visualize autophagy in starvation had been demonstrated in Fig. 4, we then operated the probe to co-stain with MitoTracker-Green to evaluate the change of mitochondria. It could be seen from Fig. 5 that the intensive red fluorescence signals of **HFI** were detected with the incubation time in HBSS. The plot profile in Fig. 5 reflected that elevated overlapped levels between the probe and MitoTracker-Green were displayed. It could be observed more intuitively at the partially enlarged view that the green fluorescence of MitoTracker-Green showed good overlap with the red fluorescence of **HFI**. These results further verified the potential use of the probe in monitoring intracellular autophagy.

**Fig. 6:**
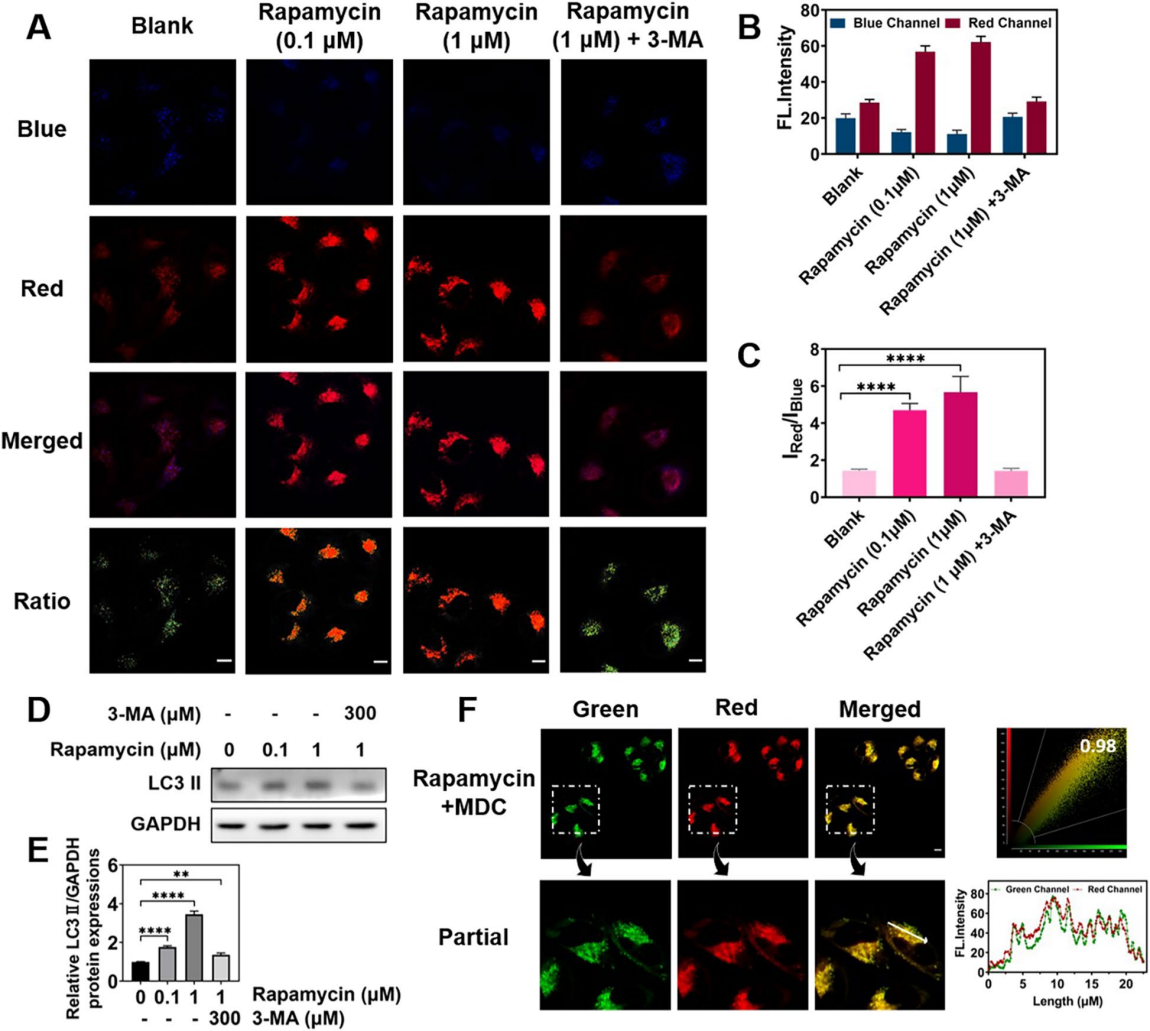
Fluorescence imaging of **HFI** in HeLa cells under rapamycin-induced autophagy conditions. **A** Fluorescence images of HeLa cells pretreated with **HFI** (10 μM) for 30 min and then incubated with rapamycin (0.1 μM or 1 μM) or rapamycin (1 μM) and 3-MA (300 μM) for another 4 h. **B** Relative fluorescence intensity for **A**. **C** The intensity ratio of the red channel to the blue channel under different induced circumstances. **D** WB illustrating the expression of LC3 II in HeLa cells. **E** Relative intensity for **D**. **F** Fluorescence imaging of HeLa cells co-stained with MDC (50 μM) and **HFI** (10 μM). Blue channel for **HFI**: *λ*_em_ = 460 ± 20 nm, *λ*_ex_ = 405 nm. Green channel for MDC: *λ*_em_ = 520 ± 20 nm, *λ*_ex_ = 405 nm. Red channel for **HFI**: *λ*_em_ = 610 ± 20 nm, *λ*_ex_ = 405 nm. Data are presented as mean ± s.d. (*n* = 3). ** *p* < 0.01 and **** *p* < 0.0001. Scale bar, 10 μm

Rapamycin, an autophagy inducer, was employed to evaluate the capability of **HFI** in indicating autophagy as well. HeLa cells were pretreated with **HFI** for 30 min and then washed with PBS buffer (pH = 7.4) three times, followed by incubation with rapamycin at different concentrations for 4 h. As depicted in Fig. 6A-C, an obvious promotion of fluorescence in the red channel was shown after the treatment with 0.1 μM rapamycin, whereas the fluorescence intensity of the blue channel reduced in correspondence, resulting in a remarkable rise of *I*_Red_/*I*_Blue_. These results implied that the probe could monitor the variation of lysosomes during autophagy. The outcomes were further magnified based on a higher concentration of rapamycin and could be blocked by the addition of 3-MA, which was consistent with the WB results (Fig. 6D-E). Afterward, the imaging accuracy of **HFI** in autophagy was specifically studied with monodansylcadaverine (MDC), a commercial autophagy tracker. Of note, the red fluorescence of the probe was immensely associated with the green fluorescence of MDC, as the Pearson’s coefficient reached up to 0.98 (Fig. 6F). Additionally, the tracking ability of HFI had also been manifested in another autophagy inducer, tamoxifen, incubated cells (Fig. S9). These results suggested the utility of HFI for tracing drug-induced autophagy.

### Evaluating autophagy in a hepatic injury model

**Fig. 7:**
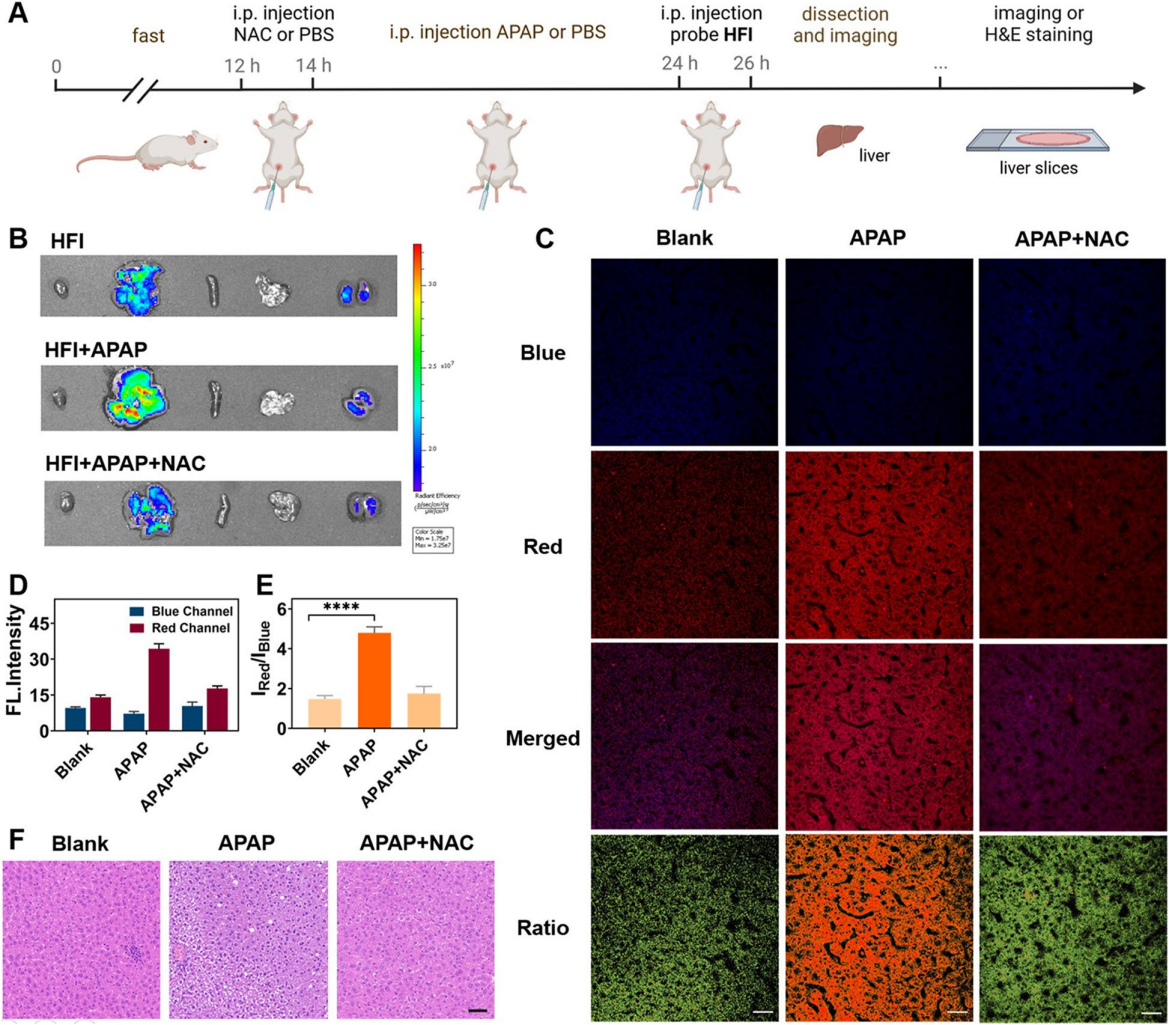
Ex vivo autophagy imaging in mice. **A** Illustration of mice model building and experiment procedure. **B** Fluorescence images of major organs of mice treated with only probe **HFI** or **HFI** and APAP or **HFI**, APAP, and NAC. **C** Confocal images of liver slices, corresponds to the liver of three different treatment methods, respectively. Scale bar, 20 μm. **D** Relative fluorescence intensity for **C**. **E** The intensity ratio of the red channel to the blue channel under different induced circumstances. Data are presented as mean ± s.d. (*n* = 3). **** *p* < 0.0001. **F** H&E staining of liver tissues of mice treated with **HFI** or **HFI** + APAP or **HFI** + APAP + NAC. Scale bar, 50 μm

With the exceptional performance of **HFI** in monitoring autophagy at cellular levels, it had been further investigated the effectiveness of the probe ex vivo. Recent studies indicate that autophagy is an important protective mechanism against APAP-induced liver injury [10, 12]. Therefore, studying the fluctuation of the autophagy levels in this disease could help to reveal the development of hepatotoxicity. In this perspective, mice were intraperitoneally treated with APAP to build a drug-induced liver injury (DILI) model (Fig. 7A). N-acetyl-L-cysteine (NAC) was provided to establish the liver protection group. As displayed in Fig. 7B, in comparison with other organs, distinct fluorescence was observed in the liver, reflecting the main distribution of the probe. Enhanced fluorescence was observed in the liver of the APAP group, whereas no significant alterations were involved in the group pretreated with NAC. Subsequently, the liver tissue sections were taken to lucubrate the details of the disease. As can be seen from Fig. 7C-D, similar weak red fluorescence was exhibited in the blank group and the ‘APAP + NAC’ group, while the fluorescence intensity of the red channel intensively increased with the induction of APAP. Conversely, compared with the other groups, a reduction of blue fluorescence was shown in the APAP group, leading to an obvious growth of *I*_red_/*I*_blue_ (Fig. 7E). These results illustrated that **HFI** could be applied in reflecting the fluctuation of autophagy in DILI. Additionally, the histological analysis of these three groups was performed by the hematoxylin and eosin (H&E) staining. According to Fig. 7F, no obvious histological changes were observed in the liver slice of the blank group, as well as the ‘APAP + NAC’ group. Nevertheless, hepatocytes undergoing watery degeneration and vacuolation were displayed under the induction of APAP. Overall, **HFI** could serve as a robust tool to investigate the process of autophagy in DILI, further promoting the evaluation of drugs for the remediation of DILI.

## Discussion

Autophagy is a highly conserved cellular process that plays a critical role in regulating numerous physiological and pathological processes. The general mechanism of autophagy involves the degradation and recycling of various cellular components to autophagic vesicles, followed by fusing with lysosomes to retrieve the recyclable contents. Since lysosomes are essential for the process of autophagy, creating fluorescent probes to monitor changes in the lysosomal microenvironment is vital for understanding the autophagic mechanism and elucidating the precise changes in autophagy levels in diseases. It has been reported that definite changes are displayed in autophagic lysosomes, such as enhanced acidification and increased viscosity. Although much effort has been put into designing probes for measuring lysosomal viscosity or pH separately, there is a need to validate the concurrent imaging of the two elements to enhance the understanding of the dynamic progression of autophagy.

In this study, we developed the first ratiometric dual-responsive fluorescent probe, **HFI**, for real-time revealing autophagic details. The synergistic promotion effect of high viscosity and low pH on the ratiometric fluorescent signal (R = *I*_610_/*I*_460_) of **HFI** enabled it to specially lit lysosomes. On account that weakly basic nitrogen-containing side chains (i.e., morpholine or N, N-dimethylethylenediamine) were not involved in the structure of **HFI**, it held the potential of the probe to image lysosomes with minimal perturbation to inherent lysosomal pH. Given the unique characteristics, **HFI** was demonstrated to employ for the detection of endogenously induced viscosity or pH variations in living cells. After that, changes in lysosomal viscosity and pH during autophagy were verified under nutrient-deprivation stress. It was found that viscosity was dramatically increased, along with a significant enhancement of acidification. Meanwhile, the capability of **HFI** to monitor intracellular autophagy induced by drugs was validated as well. More importantly, since lucubrating the molecular details of APAP-involved autophagy was still lacking, the fluctuation of the autophagy levels in this disease has been studied using **HFI** which further replenished the understanding of DILI. In a word, we have provided an effective tool for revealing autophagic details and held great promise for various biological applications.

## Conclusion

In this study, we developed a novel ratiometric fluorescent probe **HFI** that had a large Stokes shift and minimal background interference. We validated that the ratiometric fluorescent signal (R = *I*_610_/*I*_460_) of **HFI** had an excellent correlation with both viscosity and pH. Moreover, we demonstrated that **HFI** could image lysosomes with minimal perturbation to their inherent pH. These unique properties allowed us to track changes in lysosomal viscosity and pH in living cells using **HFI**. We then successfully used **HFI** to monitor intracellular autophagy induced by starvation or drugs in real-time. Importantly, **HFI** also enabled us to visualize the occurrence of autophagy in the liver tissue of a DILI model, as well as the reversible effect of hepatoprotective drugs on this event. Overall, **HFI** has great potential to serve as a useful indicator for imaging autophagic changes in viscosity and pH in complex biological samples, further assessing drug safety.

## Experimental section

Materials and apparatus, details of synthetic procedures can be found in the Supporting Information.

### Spectrometric determination

A stock solution of **HFI** was prepared in dimethyl sulfoxide (DMSO) at a concentration of 5 mM. The final concentration was used as 10 μM in the experiment unless specified. In the pH titration experiments, different pH PBS buffer were adjusted by adding 0.1 M NaOH or 0.1 M HCl solutions to give the resulting solution with different pH values. In the experiments of viscosity detection, the proportion of methanol and glycerol was adjusted to obtain the solutions with different viscosity from 2.06 cP (100% methanol) to 709 cP (100% glycerol). The ratiometric fluorescent signal (R = *I*_610_/*I*_460_) was recorded at the maximum fluorescence emission intensity of 610 nm and 460 nm. The slit width was 20 nm and 10 nm for excitation and emission separately.

### Co-localization and confocal imaging of the living cells

HeLa cells were incubated with dulbecco’s modified eagle medium supplemented with 10% fetal bovine serum, and 1% penicillin–streptomycin in an atmosphere of 5% CO_2_ and 95% air at 37 °C before use.

In the co-localization experiments, HeLa cells were cultured in the confocal dishes for 24 h under the above culture conditions. **HFI** (10 μM) was further added to the dishes for 30 min incubation. Next, the dishes were washed with PBS buffer (pH = 7.4) three times, followed by the treatment with LysoTracker Green and MitoTracker Green for another 30 min, respectively. The co-localization fluorescence images and the corresponding Pearson’s correlation coefficients were achieved by confocal laser scanning microscope (Leica TCS SP8).

For the imaging of intracellular viscosity, HeLa cells were treated with either monensin (10 μM) or nystatin (10 μM) for another 30 min. For intracellular pH calibration, the dishes were further incubated with high K^+^ buffer solutions (30 mM NaCl, 120 mM KCl, 1 mM CaCl_2_, 0.5 mM MgSO_4_, 1 mM NaH_2_PO_4_, 5 mM glucose, 20 mM HEPES, 20 mM NaOAc) at various pH values (3–7) for 5 min. Cellular fluorescence images were captured with a confocal laser scanning microscope after replacing the corresponding media with fresh media. The pseudocolor ratio images of monitoring the pH fluctuations were obtained by ImageJ software ulteriorly.

To monitor the viscosity and pH alterations during starvation, HBSS was replaced to the dishes and the fluorescence images were achieved at 0, 1, 2, 3, and 4 h incubation. The dishes containing HBSS with 3-MA (300 μM) were served as the control group. For imaging the changes in viscosity and pH in drug-induced autophagy, HeLa cells were incubated with rapamycin (0.1 μM and 1 μM) for 4 h. Meanwhile, rapamycin (1 μM) and 3-MA (300 μM) for 4 h treatment were utilized as the control groups correspondingly. MDC (50 μM) was added to the rapamycin (1 μM) dish for another 15 min incubation. The colocalization fluorescence images and the corresponding Pearson’s correlation coefficients were achieved by Leica TCS SP8 (MP + X) confocal laser scanning microscope.

### WB analysis

After incubation in HBSS or treatment with drugs (rapamycin or tamoxifen), the expression of LC3 II in HeLa cells was evaluated through WB analysis to confirm the occurrence of an autophagic process. Firstly, the treated cells were lysed with cell lysate solution for 30 min at 4 °C. Next, the protein samples were collected by the mixture with 2 × Protein Loading Buffer and boiling at 100 °C for 5–10 min. Then, the protein samples (30 μg) were separated by 15% sodium dodecyl sulfate–polyacrylamide gel electrophoresis and transferred to polyvinylidene difluoride membranes (Millipore, Massachusetts, USA). The membranes were blocked with 5% bovine serum albumin in PBST at room temperature for 1–2 h, followed by the incubation with primary antibodies for 24 h at 4 °C. Finally, the membranes were washed by PBST three times and incubated with secondary antibodies at room temperature for 1 h. Enhanced ECL Chemiluminescent Substrate Kit was used for signal detection.

### Imaging of DILI mice

The BALB/c mice (5–7 weeks) were selected to establish the DILI model. They were randomly divided into three groups: the APAP-induced group, the NAC-remediated group, and the control group. Before the experiment, the mice fasted for 12 h. For the APAP-induced group, APAP (500 mg/kg, 300 μL) was employed in the mice via intraperitoneal injection for 10 h, followed by intraperitoneal injection of **HFI** (250 μM, 100 μL) for 2 h. For the NAC-remediated group, the mice were intraperitoneally pretreated with NAC (300 mg/kg, 100 μL) for two hours before the above operation. The mice were only injected with HFI for the control group, replacing the drug injection with PBS (pH = 7.4). Then, all mice were euthanized to harvest the liver for ex vivo fluorescence imaging through IVIS Spectrum (PerkinElmer, USA). The mice’s liver tissues were prepared as sections for further bioanalysis. All animal care and experiments were carried out according to the protocols approved by the Animal Ethics and Welfare Committee, Central South University.

### Tissue histological evaluation

To evaluate acetaminophen-induced liver injury, mice were classified into three groups: intragastrically or intraperitoneally injected with acetaminophen, **HFI**, and N-acetyl-L-cysteine. All mice were humanely sacrificed and livers were excised for histological analysis via hematoxylin and eosin (H&E) staining. The fluorescence of the liver slices was further studied with a confocal laser scanning microscope.

## Supplementary Information

**Additional file 1:** **Scheme S1.** The synthesis route of **HFI**. **Fig. S1.**^ 1^H NMR spectrum of **HFI** in DMSO-*d*_6_. **Fig. S2.**^ 13^C NMR spectrum of **HFI** in DMSO-*d*_6_. **Fig. S3.** HRMS spectrum of **HFI**. **Fig. S4.** Absorption spectra (**A**) and fluorescence spectra (**B**) of **HFI **in different solvents. *λ*_ex_ = 400 nm. **Fig. S5.** Fluorescence responses of **HFI** for various analytes. Group A1: PBS, Group A2: PBS/glycerin; Group B1: PBS, Group B2: PBS/glycerin. Analytes: 1, Only probe; 2, AlCl_3_; 3, Zn(OAc)_2_; 4, FeSO_4_; 5, CoCl_2_; 6, CuCl_2_; 7, CaCl_2_; 8, Fe(NO_3_)_3_; 9, MgSO_4_; 10, HgCl_2_; 11, NaF; 12, NaBr; 13, NaI; 14, KNO_3_; 15, NaNO_2_; 16, K_2_CO_3_; 17, Na_2_SO_3_; 18, NaHS; 19, H_2_O_2_; 20, Glucose; 21, Cys; 22, GSH; 23, Lys; 24, Arg; 25, Lie; 26, Ala. C_analytes_ = 100 μM, *λ*_ex_ = 400 nm. **Fig. S6.** Cytotoxicity test for **HFI** in HeLa cells. Cell viability was measured by recording the absorbance at 490 nm. **Fig. S7. (A)** Fluorescence spectra of **HFI** in different pH PBS buffer containing an increasing proportion of glycerol. *λ*_em_ = 610 nm, *λ*_ex_ = 400 nm. (**B**) Fluorescence emission spectra of **HFI** coexisting with monensinor nystatinin methanol. *λ*_ex_ = 400 nm. **Fig. S8.** (**A**) Confocal fluorescence images of **HFI **stained HeLa cells incubated in HBSS and 3-MA for 0-4 h. (**B**) Confocal fluorescence images of **HFI** stained HeLa cells incubated in 10% FBS-contained DMEM for 0-4 h. Scale bar, 10 μm. **Fig. S9.** Fluorescence imaging of **HFI** in HeLa cells under tamoxifen-induced autophagy conditions. (**A**) Fluorescence images of HeLa cells pretreated with **HFI **for 30 min and then incubated with tamoxifenor tamoxifenand 3-MA for another 4 h. (**B**) Relative fluorescence intensity for **A**. (**C**) The intensity ratio of the red channel to the blue channel under different induced circumstances. Data are presented as mean ± s.d.. **** *p* < 0.0001. Scale bar, 10 μm. **Table S1.** An overview of recently reported fluorescent probes for viscosity and/or pH detection during autophagy.

## Data Availability

The datasets used and/or analyzed in this study are available from the corresponding author upon reasonable request.

## Abbreviations

3-MA: 3-Methyladenine
APAP: Acetaminophen
DILI: Drug-induced liver injury
DMSO: Dimethyl sulfoxide
HBSS: Hank’s Balanced Salt Solution
HFI: 2-(2-(5-(3-(Benzo[d]thiazol-2-yl)-4-hydroxyphenyl)furan-2-yl)vinyl)-1,3,3-trimethyl-3H-indol-1-ium iodide
ESIPT: Excited-state intramolecular proton transfer
HBT: 2-(2’-Hydroxy-phenyl)benzothiazole
MDC: Monodansylcadaverine
NAC: N-acetyl-L-cysteine
PBS: Phosphate buffer solution
WB: Western blot
