# Supplementary material for: Ratiometric and discriminative visualization of autophagic processes with a novel dual-responded lysosome-specific fluorescent probe
Source: Biomater Res. 2023 Jul 6;27:66. doi: 10.1186/s40824-023-00409-3 (PMC10327318; doi:10.1186/s40824-023-00409-3)
Supplement: Supplementary file 1 — Additional file 1: Scheme S1. The synthesis route of HFI. Fig. S1. 1H NMR spectrum of HFI in DMSO-d6. Fig. S2. 13C NMR spectrum of HFI in DMSO-d6. Fig. S3. HRMS spectrum of HFI. Fig. S4. Absorption spectra (A) and fluorescence spectra (B) of HFI in different solvents. λex = 400 nm. Fig. S5. Fluorescence responses of HFI for various analytes. Group A1: PBS, Group A2: PBS/glycerin; Group B1: PBS, Group B2: PBS/glycerin. Analytes: 1, Only probe; 2, AlCl3; 3, Zn(OAc)2; 4, FeSO4; 5, CoCl2; 6, CuCl2; 7, CaCl2; 8, Fe(NO3)3; 9, MgSO4; 10, HgCl2; 11, NaF; 12, NaBr; 13, NaI; 14, KNO3; 15, NaNO2; 16, K2CO3; 17, Na2SO3; 18, NaHS; 19, H2O2; 20, Glucose; 21, Cys; 22, GSH; 23, Lys; 24, Arg; 25, Lie; 26, Ala. Canalytes = 100 μM, λex = 400 nm. Fig. S6. Cytotoxicity test for HFI in HeLa cells. Cell viability was measured by recording the absorbance at 490 nm. Fig. S7. (A) Fluorescence spectra of HFI in different pH PBS buffer containing an increasing proportion of glycerol. λem = 610 nm, λex = 400 nm. (B) Fluorescence emission spectra of HFI coexisting with monensinor nystatinin methanol. λex = 400 nm. Fig. S8. (A) Confocal fluorescence images of HFI stained HeLa cells incubated in HBSS and 3-MA for 0-4 h. (B) Confocal fluorescence images of HFI stained HeLa cells incubated in 10% FBS-contained DMEM for 0-4 h. Scale bar, 10 μm. Fig. S9. Fluorescence imaging of HFI in HeLa cells under tamoxifen-induced autophagy conditions. (A) Fluorescence images of HeLa cells pretreated with HFI for 30 min and then incubated with tamoxifenor tamoxifenand 3-MA for another 4 h. (B) Relative fluorescence intensity for A. (C) The intensity ratio of the red channel to the blue channel under different induced circumstances. Data are presented as mean ± s.d.. **** p < 0.0001. Scale bar, 10 μm. Table S1. An overview of recently reported fluorescent probes for viscosity and/or pH detection during autophagy. [file 40824_2023_409_MOESM1_ESM.docx]

Supporting Information

**Ratiometric and discriminative visualization of autophagic processes with a novel dual-responded lysosome-specific fluorescent probe**

Fan Zheng ^a,b,‡^, Yeshuo Ma ^c,‡^, Jipeng Ding ^a,b^, Shuai Huang ^a,b^, Shengwang Zhang ^c^, Xueyan Huang ^a,b^, Bin Feng ^a,b^, Hongliang Zeng ^d^, Fei Chen ^a,b,*^, and Wenbin Zeng ^a,b,*^

# 1. Experimental section

## 1.1. Materials and apparatus.

All chemicals were purchased from commercial suppliers and used as received. All buffers were prepared with deionized water that had passed through a water ultra-purification system before being used. 5-Bromosalicylaldehyde, 2-Aminobenzenethiol, 2-Formylfuran-5-boronic acid, Iodomethane, 2,3,3-Trimethylindolenine, and Rapamycin were purchased from Energy Chemical Co., Ltd (Shanghai, China). Methanol (MeOH) and other organic solvents were purchased from Sinopharm Chemical Reagent Co., Ltd (Shanghai, China). Monensin and Nystatin were purchased from Topscience (Shanghai, China). ^1^H NMR (400 and 500 MHz) and ^13^C NMR (100 and 125 MHz) spectra were recorded on a Bruker Advance spectrometer (Rheinstetten, Germany) with tetramethylsilane (TMS) as an internal standard. High-resolution mass spectra (HRMS) were obtained on an Orbitrap Velos Pro LC-MS spectrometer (Thermo Scientific). UV–vis absorption spectra were measured on a UV-2450 UV-visible spectrophotometer (Shimadzu, Japan). Fluorescence spectra were recorded with a Hitachi F-2700 fluorescence spectrophotometer (Hitachi, Japan). The pH measurements were conducted with a Rex PHS-3C pH meter. Viscosity value was performed by an NDJ-8S rotary viscometer. The fluorescent cell images were acquired through a Leica TCS SP8 (MP+X) confocal laser scanning microscope (Leica, Germany).

## 1.2. Synthesis

**Scheme** **S1** The synthesis route of **HFI**.

Synthesis of Compound **3** and 1,2,3,3-tetramethyl-3H-indol-1-ium.

Compound **3** and 1,2,3,3-tetramethyl-3H-indol-1-ium were synthesized based on the reported relevant literature [1, 2].

Synthesis of **HFI**.

Compound **3** (321 mg, 1 mmol) and 1,2,3,3-tetramethyl-3H-indol-1-ium (301 mg, 1 mmol) were dissolved in 10 mL of EtOH. Piperidine (40 μL) was added dropwise, and then the mixture was reacted at 90 °C for 6 h. After the reaction was completed, the solvent was removed under reduced pressure and purified by silica column chromatography with dichloromethane/methanol (30:1, v/v) to give probe **HFI** as a dark magenta solid (460 mg, yield: 76.3%). ^1^H NMR (400 MHz, DMSO-d_6_) δ 8.81 (d, *J* = 2.3 Hz, 1H), 8.34 (d, *J* = 15.8 Hz, 1H), 8.18 (td, *J* = 9.1, 8.6, 1.6 Hz, 2H), 8.13 (dt, *J* = 8.3, 2.3 Hz, 1H), 7.86 (dt, *J* = 7.1, 1.6 Hz, 2H), 7.76 (d, *J* = 3.8 Hz, 1H), 7.62 (m, 1H), 7.60 (m, 1H), 7.58 (m, 1H), 7.52 - 7.48 (m, 1H), 7.46 (d, *J* = 3.8 Hz, 1H), 7.33 - 7.24 (m, 2H), 4.12 (s, 3H), 1.79 (s, 6H). ^13^C NMR (100 MHz, DMSO-d_6_) δ 180.46, 163.86, 160.65, 158.93, 151.83, 151.33, 143.58, 142.45, 137.12, 135.43, 130.15, 129.39, 129.10, 128.21, 126.99, 126.08, 125.66, 123.28, 122.79, 122.56, 120.52, 120.19, 118.54, 114.96, 111.01, 107.81, 51.91, 34.28, 26.13. HRMS: m/z calcd for C_30_H_25_N_2_O_2_S^+^ ([M + H]^+^) 477.1631, found 477.1700.

## 1.3. Anti-interference study

In the anti-interference study, the PBS solutions (pH = 4 or pH = 8) and the corresponding PBS/glycerin (5/5, v/v) solutions of various competitive analytes (AlCl_3_, Zn(OAc)_2_, FeSO_4_, CoCl_2_, CuCl_2_, CaCl_2_, Fe(NO_3_)_3_, MgSO_4_, HgCl_2_, NaF, NaBr, NaI, KNO_3_, NaNO_2_, K_2_CO_3_, Na_2_SO_3_, NaHS, H_2_O_2_, Glucose, Cys, GSH) were prepared as the concentration of 100 μM.

## 1.4 Cell cytotoxicity

HeLa cells were cultured in the 35 mm confocal dishes with Dulbecco’s modified eagle medium (DMEM) containing 10% fetal bovine serum (FBS), and 1% penicillin-streptomycin in an atmosphere of 5% CO_2_ and 95% air at 37 °C.

To evaluate the cytotoxicity of **HFI**, HeLa cells were evenly seeded into black 96-well plates (5×10^3^ cells/well) and allowed to finish cell adherence. Then, the original media was replaced by various concentrations of probe diluted with 10% fetal bovine serum dulbecco’s modified eagle medium (0, 1, 5, 10, 15, 20, 25, 30 μM). After 12 h, the media containing **HFI** was removed and the cells were further treated with MTT solution (10 μL/well) for 4 h. After the removal of the superfluous solution in each well, DMSO (100 μL/well) was added and the absorption at 490 nm was measured by a microplate reader (SpectraMax M2, Molecular Devices). Finally, the cell viability (%) = [(OD_sample_ - OD_blank_) / (OD_control_ - OD_blank_)] × 100%.

## 1.5. Confocal imaging of the living cells

To monitor the viscosity and pH alterations during starvation, The dishes were incubated with HBSS and 3-MA (300 μM) or 10% FBS-contained DMEM, and the fluorescence images were achieved at 0, 1, 2, 3, and 4 h incubation. They served as the control group. For the imaging of the changes in viscosity and pH in drug-induced autophagy, HeLa cells were incubated with tamoxifen (10 μM) for 4 h. Meanwhile, tamoxifen (10 μM) and 3-MA (300 μM) for 4 h treatment were utilized as the control groups correspondingly. The colocalization fluorescence images and the corresponding Pearson’s correlation coefficients were achieved by Leica TCS SP8 (MP+X) confocal laser scanning microscope.

# Reference

1. Cheng X, Huang S, Lei Q, Chen F, Zheng F, Zhong S, et al. The exquisite integration of ESIPT, PET, and AIE for constructing fluorescent probe for Hg(II) detection and poisoning. Chin Chem Lett. 2022;33:1861-4.

2. Shi J, Shu W, Tian Y, Wu Y, Jing J, Zhang R, et al. A real-time ratiometric fluorescent probe for imaging of SO_2_ derivatives in mitochondria of living cells. RSC Adv. 2019;9:22348-54.

# 2. Figures


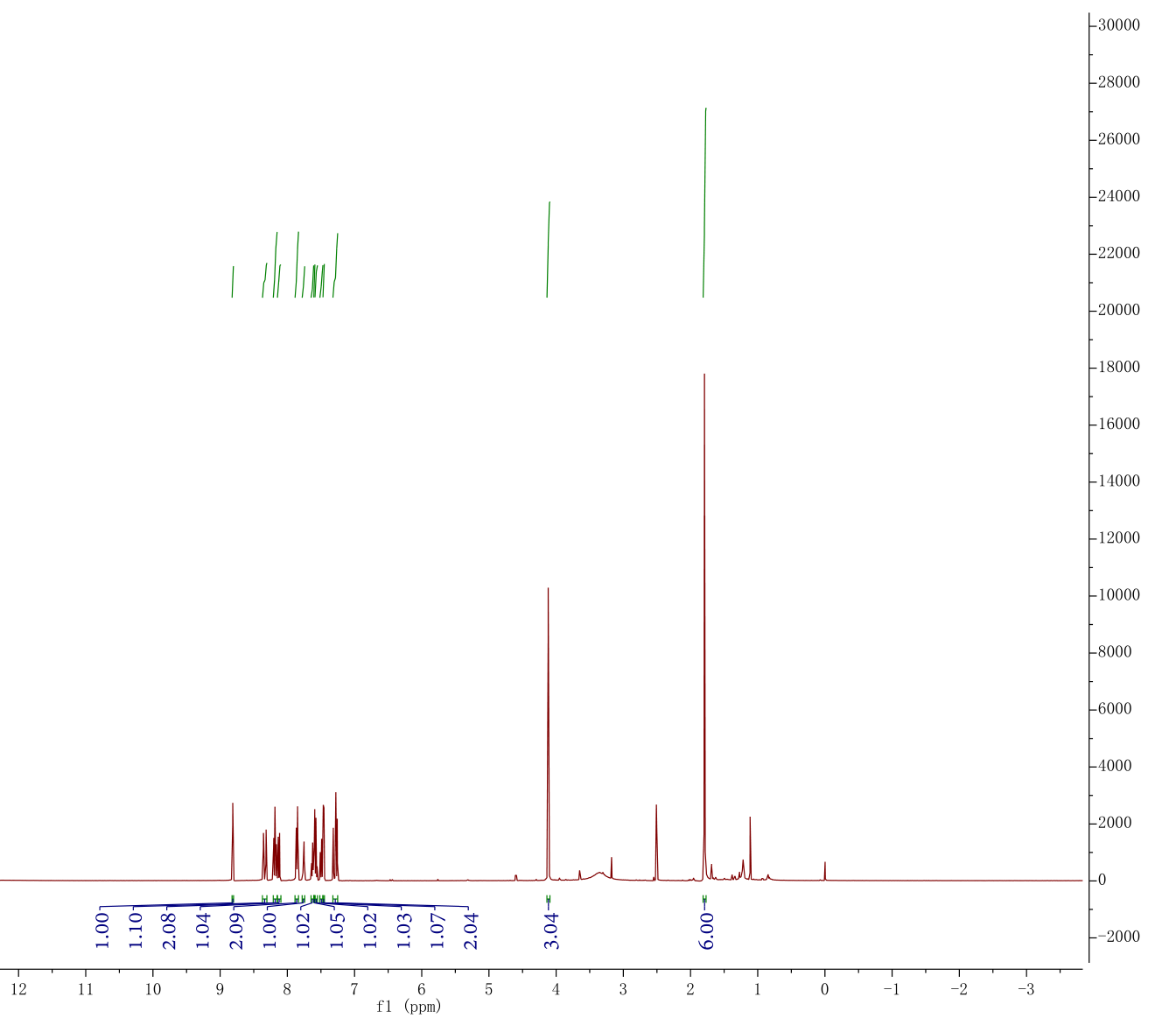


**Fig. S1.** ^1^H NMR spectrum of **HFI** in DMSO-d_6_.


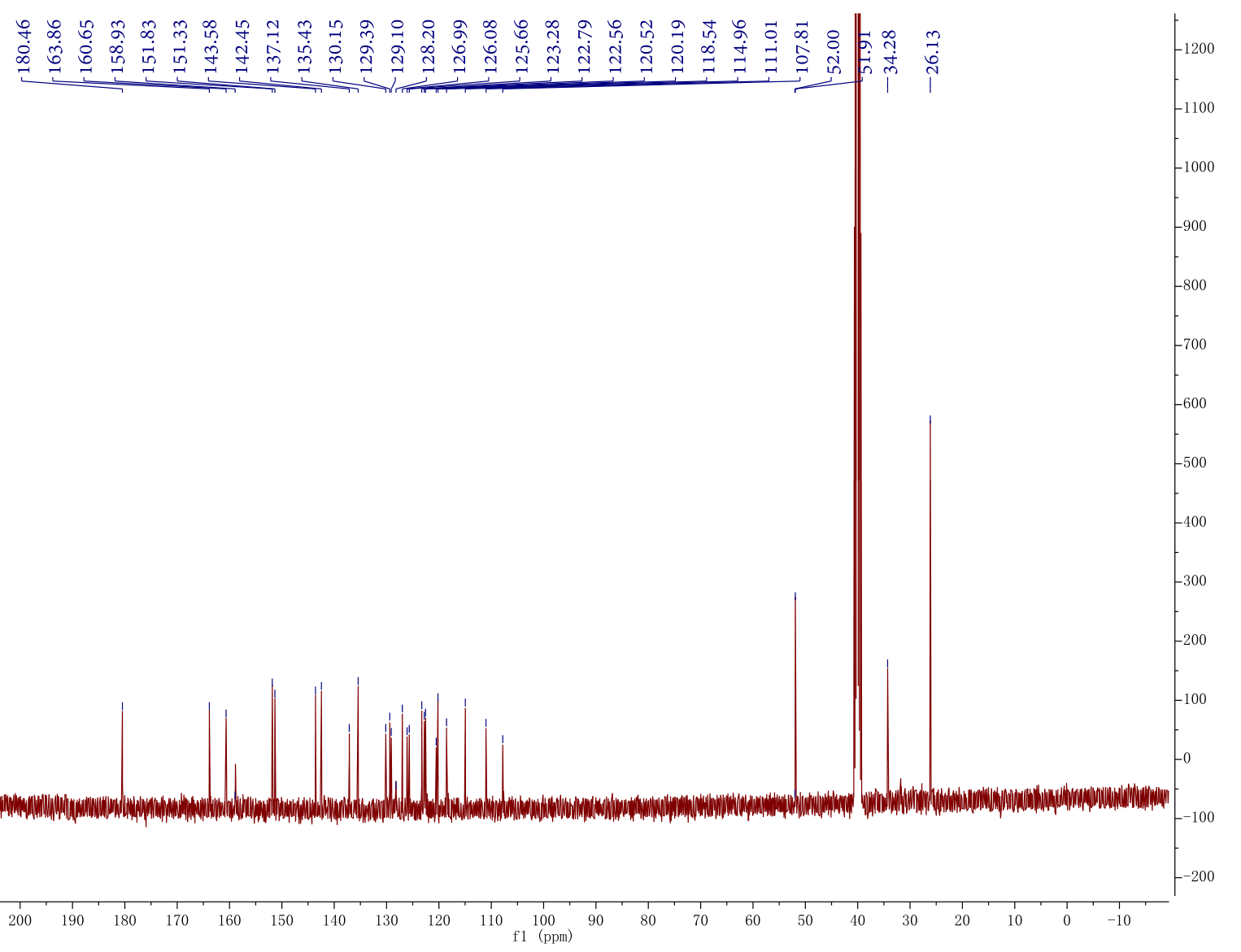


**Fig. S2.** ^13^C NMR spectrum of **HFI** in DMSO-d_6_.

**Fig. S3.** HRMS spectrum of **HFI**.


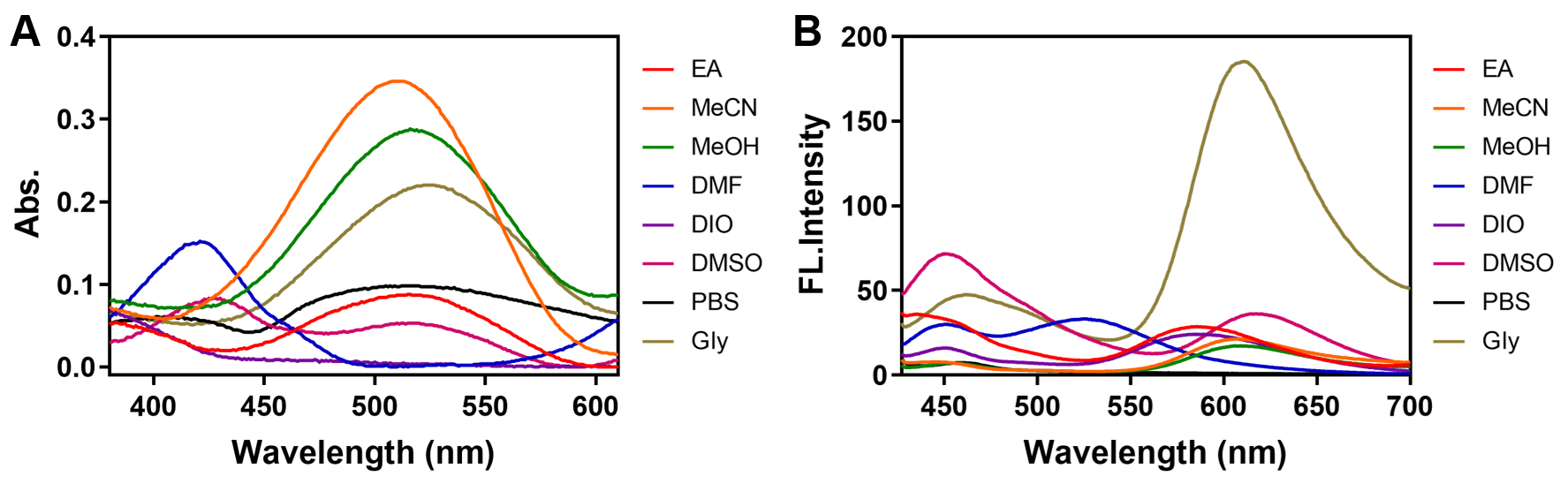


**Fig. S4.** Absorption spectra (**A**) and fluorescence spectra (**B**) of **HFI** (10 μM) in different solvents. λ_ex_ = 400 nm.


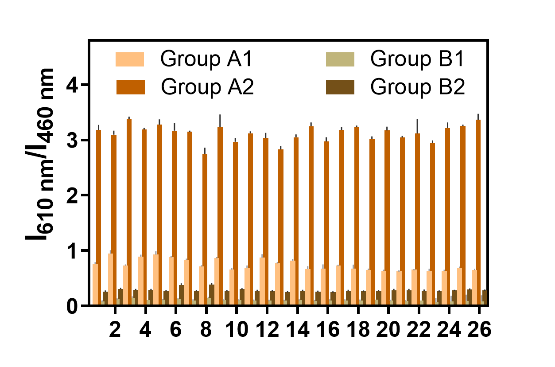


**Fig. S5.** Fluorescence responses of **HFI** (10 μM) for various analytes. Group A1: PBS (pH 4), Group A2: PBS (pH 4)/glycerin (5/5, v/v); Group B1: PBS (pH 8), Group B2: PBS (pH 8)/glycerin (5/5, v/v). Analytes: 1, Only probe; 2, AlCl_3_; 3, Zn(OAc)_2_; 4, FeSO_4_; 5, CoCl_2_; 6, CuCl_2_; 7, CaCl_2_; 8, Fe(NO_3_) _3_; 9, MgSO_4_; 10, HgCl_2_; 11, NaF; 12, NaBr; 13, NaI; 14, KNO_3_; 15, NaNO_2_; 16, K_2_CO_3_; 17, Na_2_SO_3_; 18, NaHS; 19, H_2_O_2_; 20, Glucose; 21, Cys; 22, GSH; 23, Lys; 24, Arg; 25, Lie; 26, Ala. C_analytes_ = 100 μM, λ_ex_ = 400 nm.

**
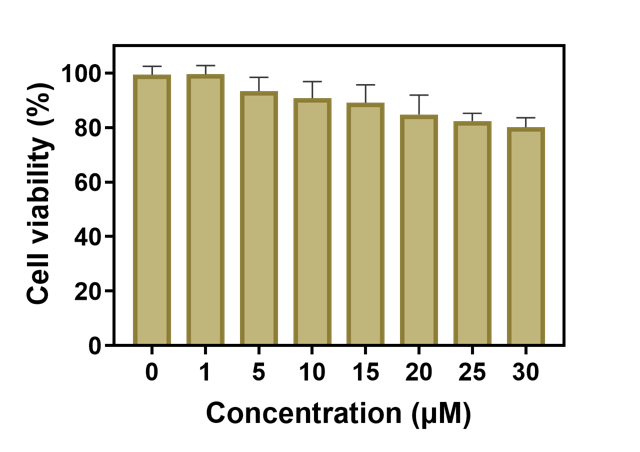
**

**Fig. S6.** Cytotoxicity test for **HFI** in HeLa cells. Cell viability was measured by recording the absorbance at 490 nm.


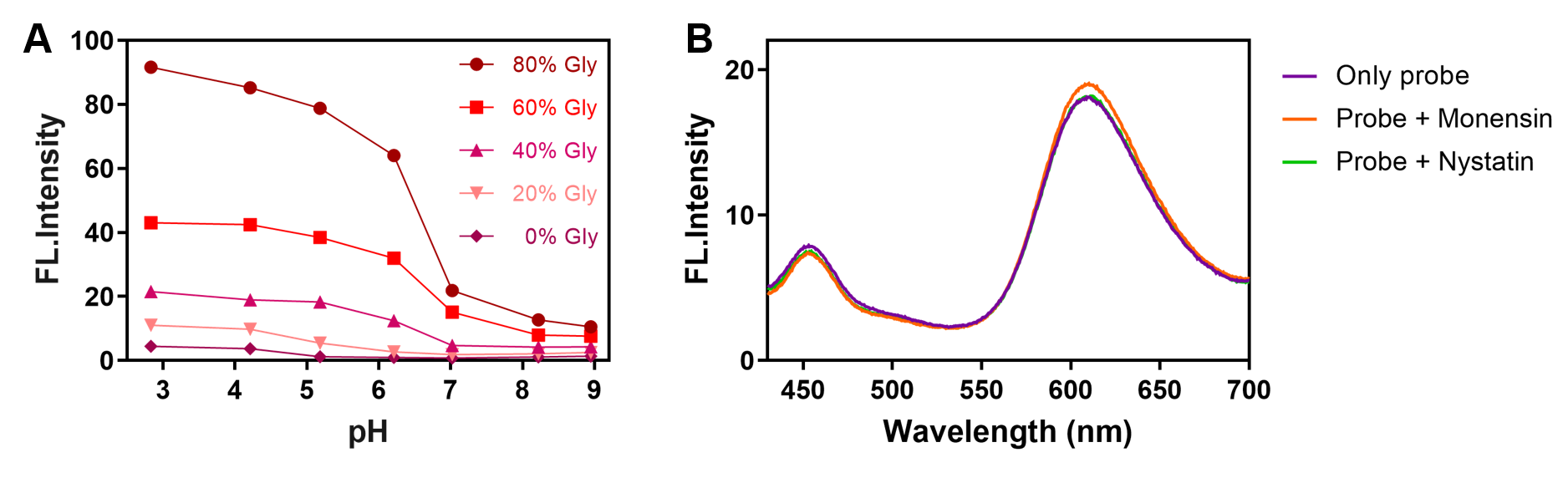


**Fig. S7.** (**A**) Fluorescence spectra of **HFI** (10 μM) in different pH PBS buffer solutions containing increasing proportion of glycerol. λ_em_ = 610 nm, λ_ex_ = 400 nm. (**B**) Fluorescence emission spectra of **HFI** (10 μM) coexisting with monensin (10 μM) or nystatin (10 μM) in methanol. λex = 400 nm.


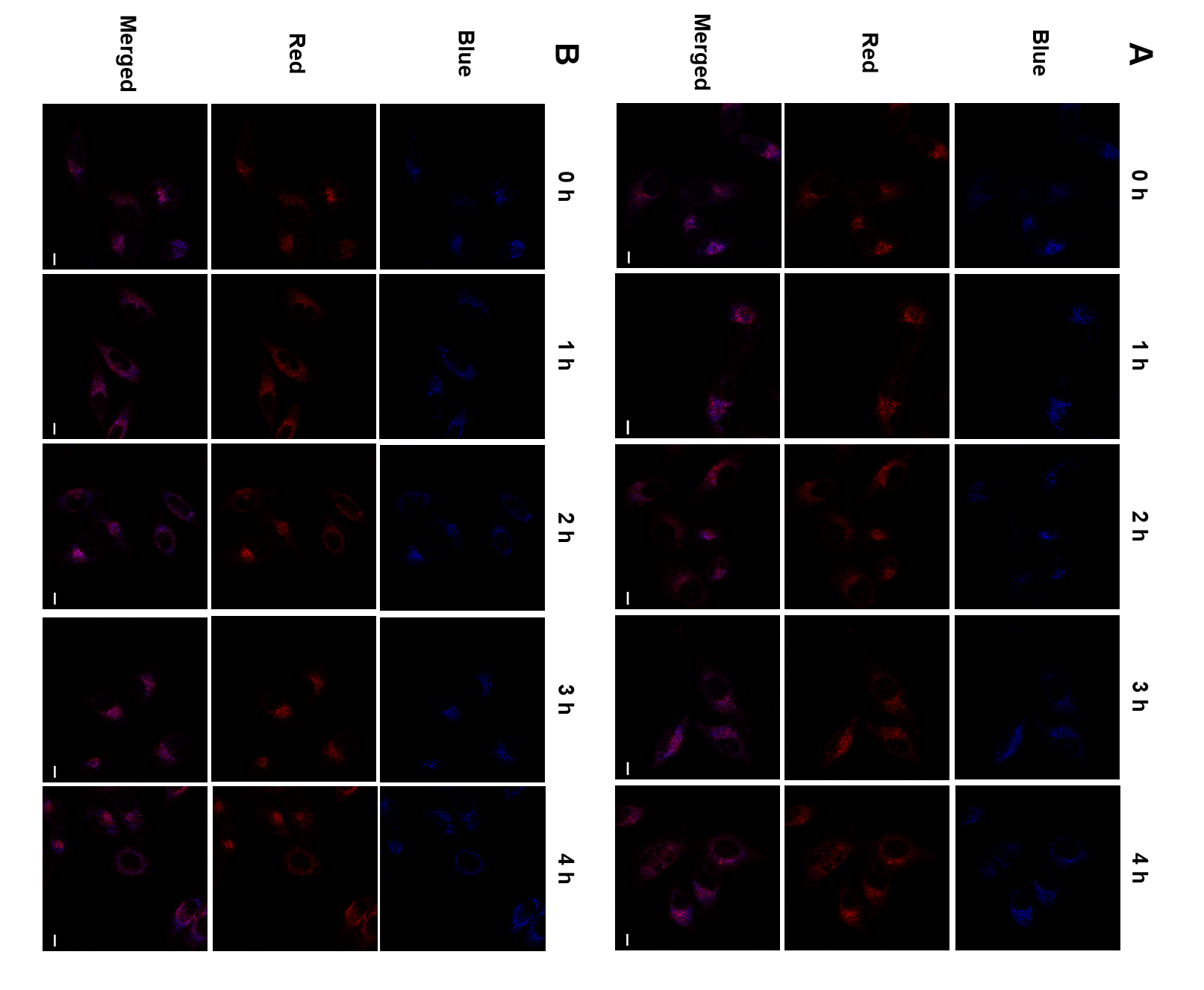


**Fig. S8.** (**A**) Confocal fluorescence images of **HFI** (10 μM) stained HeLa cells incubated in HBSS and 3-MA (300 μM) for 0-4 h. (**B**) Confocal fluorescence images of **HFI** (10 μM) stained HeLa cells incubated in 10% FBS-contained DMEM for 0-4 h. Scale bar, 10 μm.


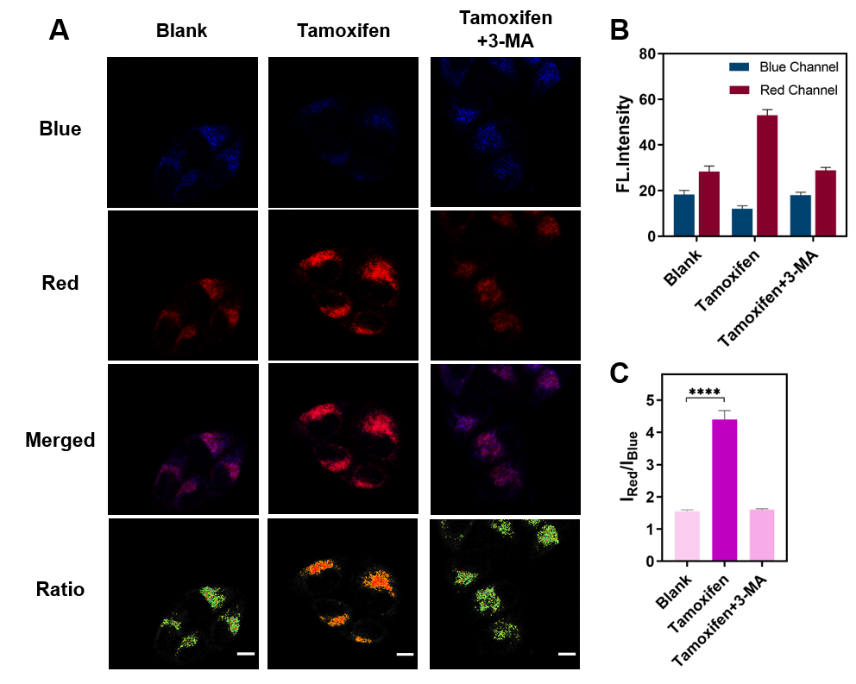


**Fig. S9.** Fluorescence imaging of **HFI** in HeLa cells under tamoxifen-induced autophagy conditions. (**A**) Fluorescence images of HeLa cells pretreated with **HFI (**10 μM) for 30 min and then incubated with tamoxifen **(**10 μM) or tamoxifen **(**10 μM) and 3-MA **(**300 μM) for another 4 h. (**B**) Relative fluorescence intensity for **A**. (**C**) The intensity ratio of the red channel to the blue channel under different induced circumstances. Data are presented as mean ± s.d. (n = 3). **** *p* < 0.0001. Scale bar, 10 μm.

# 3. Table

**Table S1**. An overview of recently reported fluorescent probes for viscosity and/or pH detection during autophagy.

| Probe | *λ*_ex_/*λ*_em_ (nm) | Stokes shift (nm) | Fluorescence enhancement in 99% glycerin | pH sensing ranges | Targeting | Reference |
| --- | --- | --- | --- | --- | --- | --- |
|  | 400/460, 610 | 210 | 15-fold | 3.5-9.0 | lysosomes | This work |
|  | 470/650 | 180 | 92-fold | × | mitochondria | [1] |
|  | 500, 820/675 | 175 | 8, 70-fold | × | lysosomes | [2] |
|  | 430/672  810/500, 680 | 242 | ~42.5-fold  - | × | lysosomes | [3] |
|  | 465/550, 597 | 132 | × | 3.0-8.0 | lysosomes | [4] |
|  | 605/780 | 175 | × | 1.0-7.0 | - | [5] |
|  | 405/510, 650 | 245 | × | 4.0-8.0 | endolysosomes | [6] |

# Reference

1. Wang X, Fan L, Wang S, Zhang Y, Li F, Zan Q, et al. Real-time monitoring mitochondrial viscosity during mitophagy using a mitochondria-immobilized near-infrared aggregation-induced emission probe. Anal Chem. 2021;93:3241-9.

2. Chai L, Liang T, An Q, Hu W, Wang Y, Wang B, et al. Near-infrared in and out: Observation of autophagy during stroke *via* a lysosome-targeting two-photon viscosity-dependent probe. Anal Chem. 2022;94:5797-804.

3. Zhai S, Hu W, Wang W, Chai L, An Q, Li C, et al. Tracking autophagy process with a through bond energy transfer-based ratiometric two-photon viscosity probe. Biosens Bioelectron. 2022;213:114484.

4. Zhang T, Huo F, Zhang W, Chao J, Yin C. Ultra-pH-sensitive sensor for visualization of lysosomal autophagy, drug-induced pH alteration and malignant tumors microenvironment. Sens Actuators B Chem. 2021;345:130393.

5. Ma Y, Liu Y, Jiang Z, Lv H, Wang J, Wang T, et al. Visualization of the pH-fluctuations in gastric ulcer living mice by the in situ near-infrared imaging. Sens Actuators B Chem. 2021;349:130747.

6. Chen R, Wang L, Ding G, Han G, Qiu K, Sun Y, et al. Constant conversion rate of endolysosomes revealed by a pH-sensitive fluorescent probe. ACS Sensors. 2023;8:2068-78.
